# Neonatal Magnesium Levels Correlate with Motor Outcomes in Premature Infants: A Long-Term Retrospective Cohort Study

**DOI:** 10.3389/fped.2014.00120

**Published:** 2014-11-05

**Authors:** Elizabeth Doll, Jacob Wilkes, Lawrence J. Cook, E. Kent Korgenski, Roger G. Faix, Bradley A. Yoder, Rajendu Srivastava, Catherine M. T. Sherwin, Michael G. Spigarelli, Erin A. S. Clark, Joshua L. Bonkowsky

**Affiliations:** ^1^Division of Pediatric Neurology, School of Medicine, University of Utah, Salt Lake City, UT, USA; ^2^Intermountain Healthcare, Salt Lake City, UT, USA; ^3^Division of Critical Care Medicine, School of Medicine, University of Utah, Salt Lake City, UT, USA; ^4^Division of Neonatology, School of Medicine, University of Utah, Salt Lake City, UT, USA; ^5^Division of Inpatient Medicine, School of Medicine, University of Utah, Salt Lake City, UT, USA; ^6^Division of Clinical Pharmacology, Department of Pediatrics, School of Medicine, University of Utah, Salt Lake City, UT, USA; ^7^Division of Maternal-Fetal Medicine, Department of Obstetrics and Gynecology, School of Medicine, University of Utah, Salt Lake City, UT, USA

**Keywords:** magnesium, prematurity, neurological, VLBW, neuroprotection, neonate

## Abstract

**Objective:** Chronic neurological deficits are a significant complication of preterm birth. Magnesium supplementation has been suggested to have neuroprotective function in the developing brain. Our objective was to determine whether higher neonatal serum magnesium levels were associated with better long-term neurodevelopmental outcomes in very-low birth weight infants.

**Study Design:** A retrospective cohort of 75 preterm infants (<1500 g, gestational age <27 weeks) had follow-up for the outcomes of abnormal motor exam and for epilepsy. Average total serum magnesium level in the neonate during the period of prematurity was the main independent variable assessed, tested using a Wilcoxon rank-sum test.

**Results:** Higher average serum magnesium level was associated with a statistically significant decreased risk for abnormal motor exam (*p* = 0.037). A lower risk for epilepsy in the group with higher magnesium level did not reach statistical significance (*p* = 0.06).

**Conclusion:** This study demonstrates a correlation between higher neonatal magnesium levels and decreased risk for long-term abnormal motor exam. Larger studies are needed to evaluate the hypothesis that higher neonatal magnesium levels can improve long-term neurodevelopmental outcomes.

## Introduction

Preterm birth can lead to a wide range of motor and intellectual disabilities affecting up to 35% of survivors (1–4). Very-low birth weight (VLBW) infants with birth weight less than 1500 g have elevated rates of cerebral palsy, epilepsy, autism, intellectual disability, and behavioral problems (5, 6). While survival rates have improved dramatically for premature infants (7), neurodevelopmental outcomes have not (8). Despite efforts to reduce preterm birth, the rate has remained relatively stable over the last few decades and was 11.7% in the United States in 2011 (9).

The neurodevelopmental problems in prematurely born infants are caused by a variety of complex pathophysiological mechanisms (10, 11) with few therapeutic options (12). Further, the complications of preterm birth are now also recognized to damage both gray matter and axon tracts and to lead to impaired neurodevelopment (13–17).

Magnesium sulfate administered antenatally has been found to reduce rates of cerebral palsy when given prior to preterm birth (18–20). Magnesium has also been found in small studies to improve neurodevelopmental outcomes in term infants with birth asphyxia (21–24). Further, several animal model studies suggest that magnesium could play neuroprotective roles in the developing vertebrate CNS (25–27).

Our hypothesis was that postnatal magnesium could serve a neuroprotective role in the developing premature brain. Magnetic resonance imaging (MRI) data suggest that longer exposure of premature infants to the extrauterine environment results in increasing impairments of CNS connectivity (28). We posited that magnesium could help protect connectivity development of premature infants, and that higher magnesium levels throughout the premature period could be neuroprotective.

To address this hypothesis, we evaluated serum magnesium levels in VLBW infants and compared these with long-term neurodevelopmental outcomes, specifically epilepsy, and abnormal motor exam. We recorded magnesium levels during the initial hospitalization, for the time period when the infants were still premature (less than 37 weeks gestation). The objective of our study was to determine whether higher neonatal magnesium levels are associated with improved long-term neurological outcomes.

## Materials and Methods

### Ethics statement

This study was approved by the Institutional Review Boards at the University of Utah and Intermountain Healthcare (IH). Data were anonymously collected and analyzed with no identifying information, and a waiver of informed consent was obtained.

### Study design and data extraction

Data extraction and analysis were performed retrospectively in a cohort of premature infants born at an IH hospital and who were seen in follow-up in the Utah State Department of Health Neonatal Follow-up Program. The cohort consisted of a consecutive series of 107 infants born between 1/1/06 through 12/31/10, with birth weights <1500 g and up to 26^6/7^ weeks gestational age (Table 1). Five patients were lost to follow-up; 27 patients had incomplete or missing data and were not included in analysis (Table 2). Serum magnesium levels were those drawn during the initial hospitalization up through the end of the premature period; defined as less than 37 weeks gestation. IH is a large, vertically integrated not-for-profit health care system in the Intermountain West encompassing 23 hospitals including the single children’s hospital. Antenatal, perinatal, and follow-up data were extracted for each patient in the cohort from the Enterprise Data Warehouse (EDW) maintained by IH.

**Table 1:** **Demographic characteristics of the study group**.

Characteristic	Study cohort *n* (%)
Gender (male)	44 (59%)
Ethnicity
Caucasian	51 (68%)
Hispanic	8 (11%)
Pacific-Islander	2 (3%)
African-American	2 (3%)
Native American	1 (1%)
Asian	3 (4%)
Unknown	8 (11%)
Multiple gestation	4 (5%)
Antenatal magnesium	20 (27%)
Gestational age (weeks)
Mean (SD; range)	25.8 (1.2; 22–27)
Median (Q1, Q3)	26.0 (25.0, 26.7)
Birth weight (g)
Mean (SD; range)	817.3 (213; 450–1410)
Median (Q1, Q3)	770 (660, 930)
Length of mechanical ventilation (days)
Mean (SD; range)	12.1 (21.6, 0–97)
Median (Q1, Q3)	3, (2, 6)

*Study group, *n* = 75*.

*SD, standard deviation; g, grams; Q1 and Q3, first and third quartiles*.

**Table 2:** **Selected demographic, birth, laboratory, and outcome characteristics of the excluded patients *n* = 27 (no magnesium levels drawn)**.

Characteristic	Excluded cohort *n* (%)
Gender (male)	9 (33%)
Ethnicity
Caucasian	20 (71.4%)
Hispanic	3 (11%)
Pacific-Islander	0 (0%)
African-American	1 (3.6%)
Native American	0 (0%)
Asian	0 (0%)
Unknown	3 (14%)
Multiple gestation	4 (15%)
Gestational age (weeks)
Mean (range)	25.7 (23.8–27)
Birth weight (g)
Average	789
Range	500–1110
Length of mechanical ventilation (days)
Mean (range)	8.6 (0–45)

We queried the EDW using unique identifiers assigned to each of the cohort infants for the period including up to 5 years after birth. Data collected from the EDW included name; date of birth; gender; ethnicity; birth weight; birth head circumference; gestational age; presence of multiple gestation; administration of corticosteroids prior to delivery; administration of magnesium sulfate prior to delivery; mode of delivery; length of hospitalization; all neonatal total serum magnesium levels; days requiring mechanical ventilation; and the presence of seizures (ICD-9 codes 779.0 and 345.x). Of note, diagnoses of seizures at any time during the NICU hospitalization were excluded, as was the diagnosis of febrile seizures. Data extracted manually from the neurodevelopmental assessment at age 20–36 months included neurological exam for hypotonia, spasticity, and/or cerebral palsy.

Long-term follow-up for infants was assessed and recorded using two sources. First, for the outcome of the abnormal motor exam, data from the Utah State Department of Health Neonatal Follow-up Program were obtained. A standardized neurological motor exam was performed by a developmental pediatrician or pediatric neurologist when the infant was between 20 and 36 months of age. An abnormal motor exam was defined as cerebral palsy (including hypertonia/spasticity or dystonia), hypotonia, or spasticity. Second, for the outcome of epilepsy, we followed the infants for up to 5 years after birth using the EDW. We defined epilepsy as any encounter that had a record of the patient having had a seizure and for which the patient was placed on an anti-epileptic drug. The outcome of epilepsy excluded seizures that occurred solely during the NICU hospitalization; and febrile seizures.

We limited the time period of magnesium levels used in our analysis to that of prematurity only; i.e., less than 37 weeks gestation. In the IH system, the lower and upper limits of magnesium levels are defined as from 1.2 to 2.8 mg/dL, respectively.

### Statistical analysis

Statistical analyses were performed using SAS Analytics Pro version 9.3 (SAS Inc.). Descriptive statistics were used to characterize the study cohort. Wilcoxon Rank-Sum tests were used to compare magnesium levels for the outcomes of seizures and of composite abnormal motor exams. An alpha level of 0.05 was used to determine statistical significance; *p*-values were two-sided. For multivariate logistic regression analysis we modeled birth weight and magnesium levels by analysis of quartiles.

## Results

We collected all total serum magnesium levels (*n* = 223) drawn on a cohort of 75 very-low birth weight (VLBW) infants during their initial, post-birth hospitalization (Table 1). There were no deaths in the study cohort. On average infants had their magnesium levels checked three times, but the number of magnesium levels checked ranged from 1 to 17 times (Table 3). Average total serum magnesium level was 2.4 mg/dL with a range of 1.1–5.8 mg/dL.

**Table 3:** **Characteristics of serum magnesium testing**.

Characteristic
Average number of draws/infant	3
Range number of draws/infant	1–17
Age at first draw (DOL) (avg.)	3.5
Range of age for first draw (DOL)	0–56
Proportion of draws <3 DOL	36%
Serum levels (mg/dl)
Mean (SD; range)	2.4 (0.83; 1.1–5.8)
Median	2.2 (1.9, 2.7)
Mode	2.1

*DOL, day of life; SD, standard deviation*.

There were 10 (13%) infants that had epilepsy, and 24 (32%) with abnormal motor exams. Abnormal motor exam, which was defined as the presence of cerebral palsy, hypotonia, or spasticity, was assessed between 20 and 36 months after birth. The outcome of epilepsy was assessed for by following patient outcomes for up to 5 years after birth. Importantly, to avoid inflating epilepsy rates, “epilepsy” was defined for the purpose of outcomes by excluding seizures which occurred in the neonatal period only, and by excluding febrile seizures.

**Figure 1:**
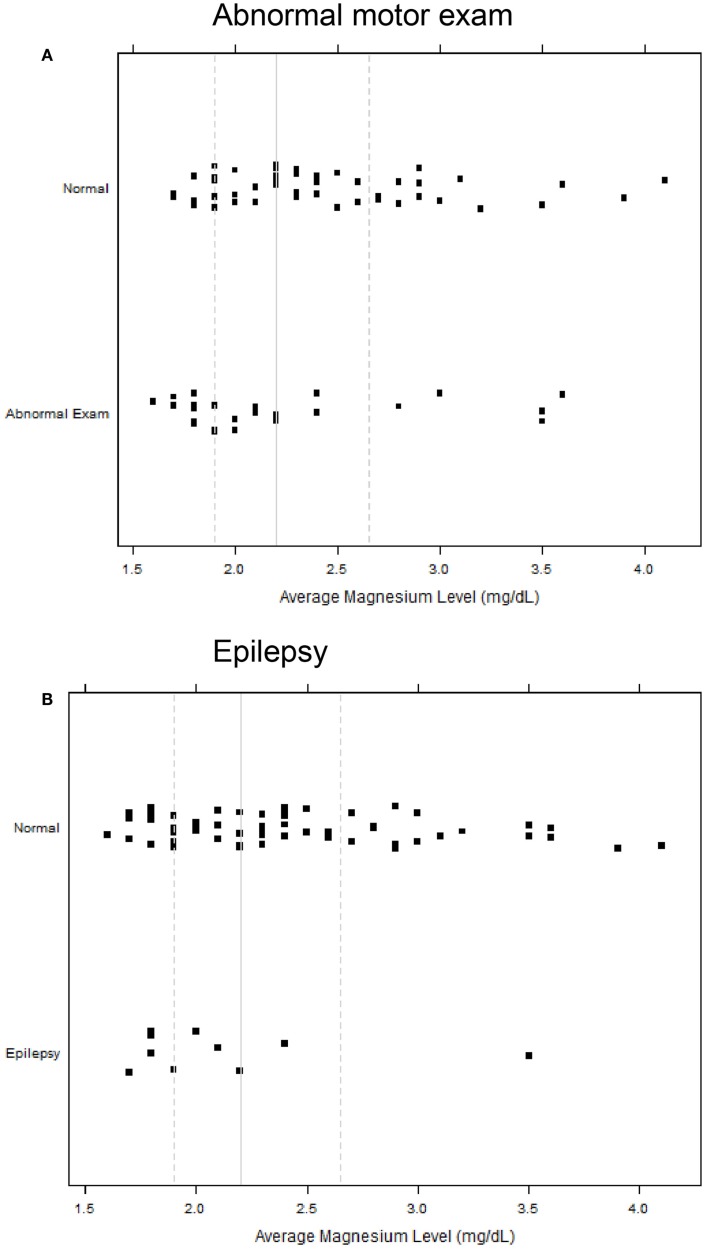
**Strip scatterplots of average magnesium levels (*x*-axis) and neurodevelopmental outcomes, for abnormal motor exam (A), and epilepsy (B)**. Thin line is the median; dotted lines show 25th and 75th quartiles.

All infants with epilepsy also had an abnormal motor exam. We found that children with abnormal motor exams had statistically significant lower magnesium levels in the neonatal period (*p* = 0.037) (Table 4; Figure 1A). Infants who went on to develop epilepsy had lower average minimum magnesium levels in the neonatal period, but this was not statistically significant (*p* = 0.060) (Table 4; Figure 1B). We also performed logistic regression analyses that included birth weight and magnesium level. While they showed a trend toward lower magnesium levels associated with increased risk for abnormal motor outcome or for epilepsy, the sample size was underpowered and did not show statistically significant differences (Table 5).

**Table 4:** **Wilcoxon rank-sum tests for epilepsy and for abnormal motor exam**.

Magnesium level	Outcome		*p*-Value
**Epilepsy**			
	No (*n* = 65)	Yes (*n* = 10)	
Median	2.30	1.95	0.060
**Abnormal motor exam**			
	No (*n* = 51)	Yes (*n* = 24)	
Median	2.30	2.00	0.037

*The number of patients with the outcome is indicated in parentheses. Magnesium levels are milligrams per deciliter. Two-sided *p*-values are given*.

**Table 5:** **Logistic regression results; (A) Multivariable regression analysis for the outcome of seizures, analyzed for birth weight and magnesium levels; (B) Multivariable regression analysis for the outcome of abnormal motor exam, analyzed for birth weight, and magnesium levels**.

**(A)**				
**Average magnesium level quartile by seizures**				
Quartile of Mg level	No	Yes		
Q1, Mg level ≤1.9	17 (77%)	5 (23%)		
Q2, 1.9 < Mg level ≤2.2	14 (82%)	3 (18%)		
Q3, 2.2 < Mg level ≤2.7	18 (95%)	1 (5%)		
Q4, Mg level > 2.7	16 (94%)	1 (6%)		
**Birth weight quartile by seizures**				
Quartile of Mg level	No	Yes		
Q1, Birth weight ≤660	19 (95%)	1 (5%)		
Q2, 660 < Birth weight ≤770	14 (78%)	4 (22%)		
Q3, 770 < Birth weight ≤930	16 (84%)	3 (16%)		
Q4, Birth weight > 930	16 (89%)	2 (11%)		

**Parameter**	**Estimate**	**LCL**	**UCL**	***p*-Value**

**Birth weight (Q4 = Ref)**				
Q1	1.00	0.22	4.51	1.00
Q2	1.34	0.31	5.89	0.696
Q3	1.15	0.28	4.76	0.842
**Milligrams level (Q4 = Ref)**				
Q1	2.40	0.62	9.24	0.842
Q2	1.30	0.30	5.61	0.725
Q3	0.28	0.05	1.72	0.169

**(B)**				
**Average magnesium level quartile by abnormal motor scores**				
Quartile of milligram level	No	Yes		
Q1, Mg level ≤1.9	11 (50%)	11 (50%)		
Q2, 1.9 < Mg level ≤2.2	11 (65%)	6 (35%)		
Q3, 2.2 < Mg level ≤2.7	17 (89%)	2 (11%)		
Q4, Mg level >2.7	12 (71%)	5 (29%)		
**Birth weight quartile by abnormal motor scores**				
Quartile of milligrams Level	No	Yes		
Q1, Birth weight ≤660	17 (77%)	5 (23%)		
Q2, 660 < Birth weight ≤770	14 (82%)	3 (18%)		
Q3, 770 < Birth weight ≤930	18 (95%)	1 (5%)		
Q4, Birth weight >930	16 (94%)	1 (6%)		

**Parameter**	**Estimate**	**LCL**	**UCL**	***p*-Value**

**Birth weight (Q4 = Ref)**				
Q1	0.61	0.05	7.77	0.700
Q2	3.10	0.45	21.39	0.251
Q3	1.46	0.20	10.50	0.707
**Milligrams level (Q4 = Ref)**				
Q1	4.82	0.48	47.91	0.180
Q2	3.51	0.31	39.89	0.311
Q3	0.86	0.047	15.59	0.917

*LCL, 95% lower confidence limit; UCL, 95% upper confidence limit; Q1, first quartile*.

*(A) Fisher’s exact *p*-value = 0.316; Fisher’s exact *p*-value = 0.891*.

*(B) Fisher’s exact *p*-Value = 0.058; Fisher’s exact *p*-value = 0.434*.

We also considered whether there might be potential clinical history confounders, by examining whether there were other clinical characteristics associated with high or low magnesium levels (high defined as 2.3 mg/dL or above, low 2.2 mg/dL and below) (Table 6), and by analyzing whether other clinical characteristics were associated with the outcomes of epilepsy or abnormal motor outcome (Table 7). While there were some differences between the groups, there was not a consistent trend.

**Table 6:** **Comparison of clinical variables in “low” and “high” magnesium patient groups**.

Clinical variable	Low magnesium	High magnesium
Male gender	57%	59%
Caucasian	71%	68%
Birth weight	872 g	755 g
Gestational age	25.9 weeks	25.7 weeks
Maternal age	27.5 years	28.4 years
Maternal parity	3.1	2.6
Maternal smoking	29%	14%
Medicaid	36%	38%
Maternal diabetes	0	5.4%
Maternal hypertension	7.1%	32%
Maternal drug use	7.1%	0
Multiple gestation	0	10.8%
Antepartum hemorrhage	29%	16%
Chorioamnionitis	4.8%	2.7%
Steroids pre-delivery	83%	73%
Ventilator days	12.3	10.9
ECMO	0%	0%
Hydrocephalus	7.1%	8.1%
NEC	17%	11%
IVH (Grade II, III, IV)	40%	27%
Length of stay	109 days	104 days

*“Low” magnesium was defined as average magnesium level of 2.2 mg/dL or lower; “high” was defined as 2.3 mg/dL or higher*.

*ECMO, extra-corporeal membrane oxygenation; IVH, intraventricular hemorrhage; NEC, necrotizing enterocolitis*.

**Table 7:** **Comparison of clinical variables and the outcomes for epilepsy or abnormal motor exam**.

Clinical variable	Epilepsy	Abnormal motor
	+/−	+/−
Male gender	90/57%	65/55%
Caucasian	60/71%	69/70%
Birth weight	832/815 g	833/810 g
Gestational age (days)	179/181	180/181
Maternal age	25.5/28.3	25.7/29
Maternal parity	4.2/2.7	2.47/1.74
Maternal smoking	10/23%	12/26%
Medicaid	60/33%	46/32%
Maternal diabetes	0/2.9%	0/3.8%
Maternal hypertension	0/21.7%	12/23%
Maternal drug use	0/4.3%	0/5.7%
Multiple gestation	0/5.8%	0/7.5%
Antepartum hemorrhage	50/19%	31/19%
Chorioamnionitis	0/4.3%	3.8/5.7%
Mg pre-delivery	20/35%	19/28%
Steroids pre-delivery	90/77%	88/74%
Ventilator days	19/11	17/9.2
ECMO	0/0%	0/0%
Hydrocephalus	20/5.8%	15/3.8%
NEC	10/14%	12/15%
IVH (Grade II, III, IV)	50/32	50/26
Length of stay (days)	132/103	123/98

*+, diagnosis present; −, diagnosis absent (e.g., normal)*.

*ECMO, extra-corporeal membrane oxygenation; IVH, intraventricular hemorrhage; NEC, necrotizing enterocolitis*.

## Discussion

We examined the correlation between postnatal magnesium levels and neurodevelopmental outcomes in premature infants. While prenatal magnesium administration has been associated with a decreased risk of cerebral palsy (18–20), our study demonstrates that postnatal serum magnesium levels in VLBW infants may be associated with improved neurodevelopmental outcomes. Specifically, higher serum magnesium levels in preterm infants were associated with lower rates of an abnormal motor exam (spasticity, cerebral palsy, or hypotonia). There was a lower risk for epilepsy, but this finding was not statistically significant.

Our study raises the possibility that there may be a broader window of opportunity for magnesium administration in premature infants to help improve neurodevelopmental outcomes. That is, perhaps magnesium supplementation could also be considered in premature infants and not only in mothers at risk for preterm birth. Use of magnesium sulfate would have to be balanced with concerns for potential adverse side-effects in premature infants (29, 30). Another issue requiring further study will be research on the mechanism(s) of potential neuroprotection. This is because most infants had a magnesium level in the “normal” range, and because there was significant overlap in the magnesium levels of infants with normal outcomes compared to infants who developed epilepsy or abnormal motor outcomes (Figure 1).

Animal model data show a neuroprotective role for magnesium against injury in the developing CNS (25–27). Further, in term infants with birth asphyxia a potentially protective role has been suggested for magnesium (21–24). There are multiple potential mechanism(s) by which magnesium could exert neuroprotective effects. Magnesium can block calcium influx through the *N*-methyl-d-aspartate (NMDA) receptor channel and thereby reduce glutamate excitotoxicity; can reduce inflammatory cytokine and free radical production; can stabilize membranes; and can normalize blood pressure fluctuations (31–33). Magnesium can also prevent activation of the hypoxia inducible factor 1α (HIF1α) pathway that leads to axon pathfinding errors (27).

Limitations of this study included sample size, retrospective data collection, and missing follow-up for some infants. While the presence of an abnormal neuro-motor exam can be determined by age 20 months as was done in this study, a more extensive longitudinal study with objective scoring, such as using the Bailey Scale of Infant Development, would provide more reliable outcomes data. We excluded 32 infants from our analysis because of incomplete data, including a lack of magnesium levels. The small sample size also led to limitations on performing multivariate regression analyzes (34). Another bias could arise because timing of magnesium level blood draws were not evenly distributed in the different infants; and were not distributed evenly across the hospitalization. Because of the sample size, we were not able to control for multiple factors that could play an important role in the outcomes, such as intraventricular hemorrhage; maternal steroid or magnesium administration; or birth weight, among others. Pharmacokinetics and pharmacodynamics of serum magnesium can be affected by both endogenous and iatrogenic factors, including medications and calcium metabolism. Our study did not address these issues. In fact, normative premature infant magnesium levels, and effects of maternal magnesium administration on neonatal levels, are under active study (35, 36), and are important subjects for future research.

Our pilot findings raise the possibility that magnesium levels during a critical developmental time window could affect neurological outcome. In the U.S. 500,000 births each year are premature, while worldwide it is estimated that 12.9 million infants yearly are born before 37 weeks gestation (9, 37, 38). This significant burden of prematurity, with its attendant risks for adverse neurodevelopmental outcomes, warrants further investigations into potential neuroprotective roles for magnesium after preterm birth.

## Conflict of Interest Statement

The authors declare that the research was conducted in the absence of any commercial or financial relationships that could be construed as a potential conflict of interest.

## Abbreviations

CNS, central nervous system; EDW, enterprise data warehouse; IH, intermountain healthcare; VLBW, very-low birth weight.
